# Postoperative microcystic meningioma of the fourth ventricle with subsequent giant cell reparative granuloma: a case report

**DOI:** 10.3389/fonc.2025.1624498

**Published:** 2025-08-06

**Authors:** Fangfang Xu, Chengzhi Fu, Qian Li, Fei Dong, Jinlong Tang, Chao Wang, Chongran Sun

**Affiliations:** ^1^ Department of Radiology, The Second Affiliated Hospital, Zhejiang University School of Medicine, Hangzhou, China; ^2^ Department of Radiology, The First Affiliated Hospital, Three Gorges University School of Medicine, Yichang, China; ^3^ Department of Pathology, The Second Affiliated Hospital, Zhejiang University School of Medicine, Hangzhou, China; ^4^ Department of Neurosurgery, The Second Affiliated Hospital, Zhejiang University School of Medicine, Hangzhou, China

**Keywords:** microcystic meningioma, giant cell reparative granuloma, magnetic resonance imaging, fourth ventricle, case report

## Abstract

**Background:**

Microcystic meningioma (MM) is a distinctive benign tumor typically located in the supratentorial region. Giant cell reparative granuloma (GCRG) is another rare reactive benign lesion associated with surgical trauma or tissue injury. The occurrence of MM in the fourth ventricle is extremely uncommon, and the development of GCRG following cranial tumor surgery is rare.

**Case report:** We present a case of MM extending into the fourth ventricle in a 54-year-old man. The initial diagnosis was based on magnetic resonance imaging (MRI), and the tumor was successfully treated with surgery. Postoperative histopathological analysis confirmed the diagnosis of MM. However, a mass was detected at the original surgical site during a follow-up examination one year later. Combined preoperative imaging and postoperative pathology confirmed the final diagnosis of giant cell reparative granuloma (GCRG).

**Conclusion:**

In cases of MM at atypical locations and GCRG, an imaging-based differential diagnosis is crucial for guiding treatment decisions and predicting prognosis. Regular postoperative reviews are also essential for detecting complications.

## Introduction

Microcystic meningioma (MM) is a rare benign tumor of the meningioma epithelium, accounting for approximately 1.6% of all intracranial meningiomas. It is characterized by myxomatous, microcystic, and vacuolated histomorphology and typically occurs in the supratentorial region (1). To our knowledge, only one case of MM occurring in the fourth ventricle has been reported in a dog, and no such cases have been documented in humans (2). Due to the extreme rarity of MM in the fourth ventricle, preoperative diagnosis remains highly challenging. Giant cell reparative granuloma (GCRG) is another rare benign lesion, generally regarded as a reactive proliferation arising in response to surgical trauma or tissue injury (3).

Herein, we present the first reported case of MM in the fourth ventricle of a man, with the subsequent development of GCRG identified one year after surgery. This report provides a comprehensive overview of the radiological characteristics, diagnostic considerations, and therapeutic management associated with these rare lesions, contributing to improved diagnostic recognition.

## Case report

**Figure 1:**
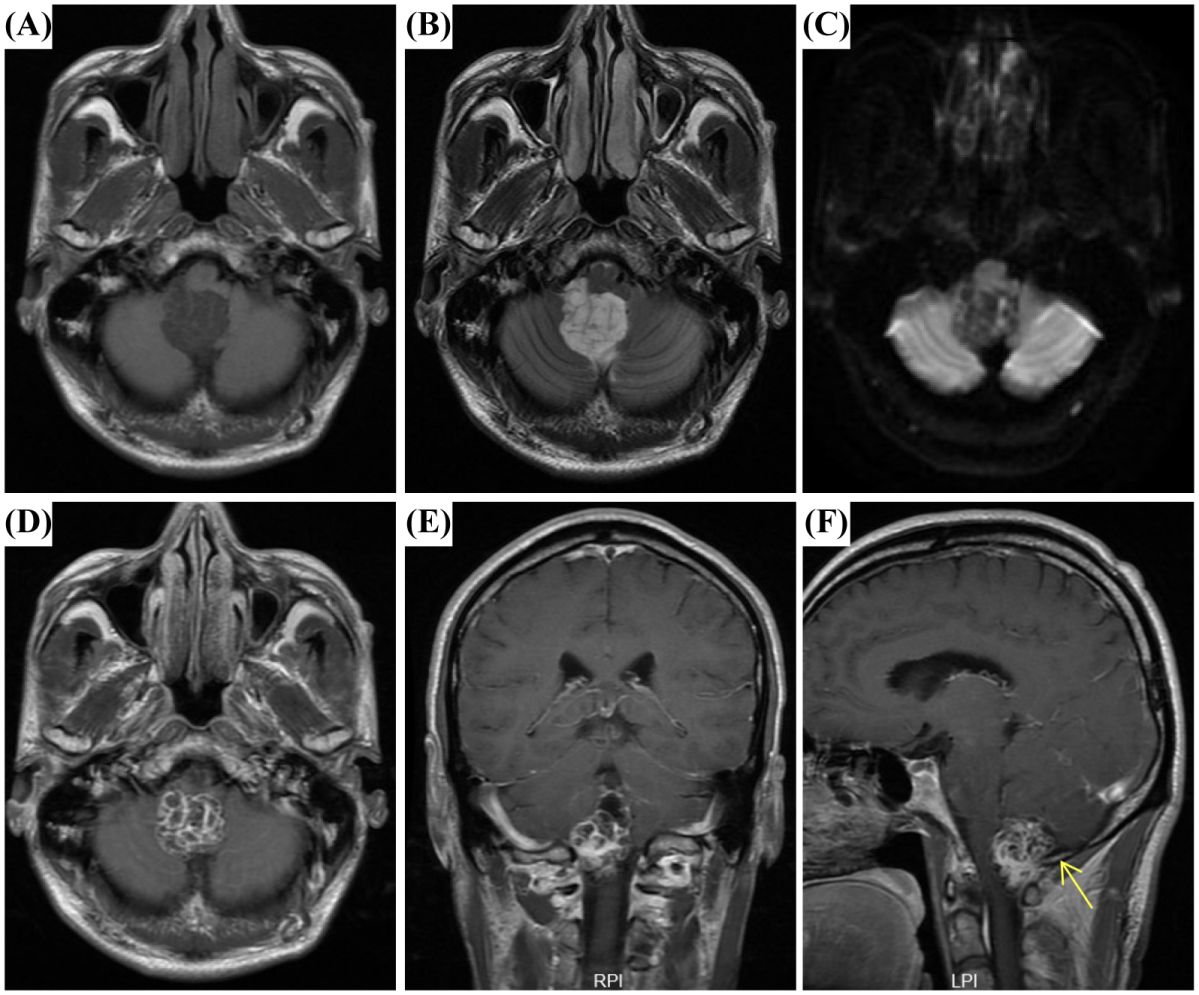
Magnetic resonance imaging (MRI) findings of Microcystic meningioma (MM). **(A)** Axial T1-weighted imaging (T1WI) showed a slightly hypointense cyst wall and hypointense content. **(B)** Axial T2-weighted imaging (T2WI) demonstrated a hypointense wall and hyperintense content with clear boundaries. **(C)** Diffusion-weighted imaging (DWI) revealed a hyperintense cyst wall and hypointense content. **(D, E)** Post-contrast axial and coronal T1WI displayed a lobulated, heterogeneously enhancing mass with a characteristic “soap-bubble” or microcystic appearance. **(F)** Post-contrast sagittal T1WI showed heterogeneous enhancement with a dural tail sign (arrows).

A 54-year-old man with a three-year history of paroxysmal headaches presented to our hospital. Physical examination revealed that he was alert and oriented, with fluent speech; pupils were equal and reactive to light; limb muscle strength and tone were normal; gait was stable; and the Romberg sign was negative. He had no significant past surgical or relevant family history. Further laboratory tests revealed an elevated carcinoembryonic antigen (CEA) level of 7.5 ng/mL (reference range: <5 ng/mL), while all other parameters, including complete blood count, renal function, and liver function tests, were normal. Subsequently brain magnetic resonance imaging (MRI) revealed a well-defined multilocular cystic lesion in the fourth ventricle. T1-weighted imaging (T1WI) showed a slightly hypointense cyst wall and hypointense content (**Figure 1A**). T2-weighted imaging (T2WI) demonstrated a hypointense wall and hyperintense content with clear boundaries (**Figure 1B**). Diffusion-weighted imaging (DWI) revealed a hyperintense wall and hypointense content (**Figure 1C**). Post-contrast axial and coronal T1WI displayed a lobulated, heterogeneously enhancing mass with a characteristic “soap-bubble” or microcystic appearance (**Figures 1D, E**). Notably, post-contrast sagittal T1WI showed heterogeneous enhancement along with a dural tail sign (**Figures 1F**). These features suggested a preoperative diagnosis of fourth ventricular ependymoma.

**Figure 2:**
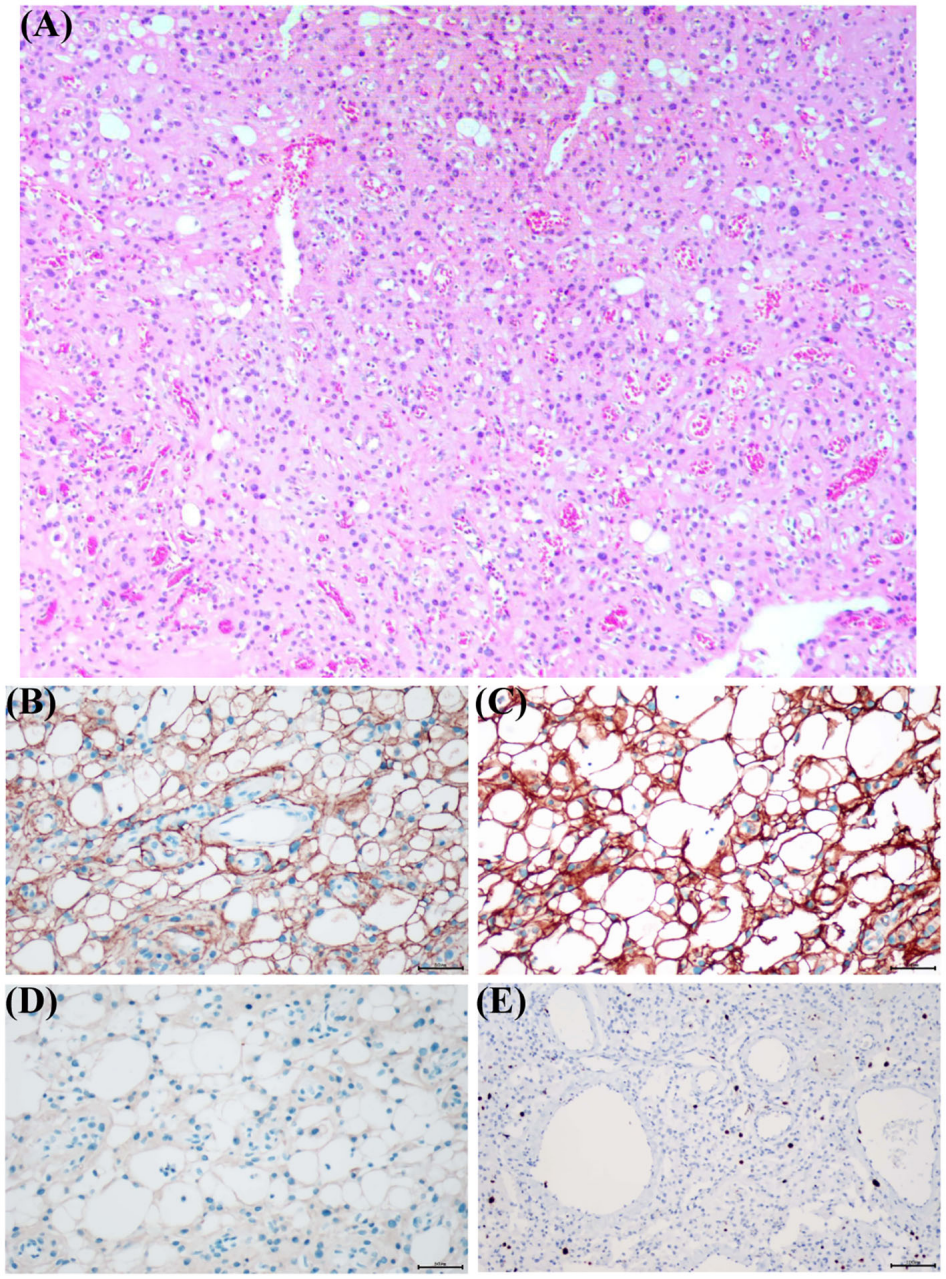
Pathological findings of MM. **(A)** Histopathological examination (HE) staining of the resected specimen showed moderate cell density, mild to moderate pleomorphism, abundant vasculature, vitreous degeneration, and both microcystic and large cystic changes. **(B–E)** Immunohistochemical (IHC) analysis showed positive for epithelial membrane antigen (EMA) (magnification, ×200) and SSTR2 (magnification, ×200), and negative for inhibin (magnification, ×200). **(E)** The Ki-67 proliferation index was low (5–10%) (magnification, × 100).

After excluding contraindications and obtaining informed consent from the patient and his family, the fourth ventricular tumor was completely resected through the posterior median approach. Intraoperatively, the mass appeared polycystic, light red, and partially adherent to the surrounding brain tissue, measuring approximately 23 × 40 × 27 mm. Histopathological examination (HE) revealed a tumor with moderate cellularity, mild to moderate pleomorphism, abundant vasculature, vitreous degeneration, and both microcystic and large cystic changes (**Figure 2A**). The nuclei were enlarged and hyperchromatic, with occasional prominent nucleoli and eosinophilic cytoplasm. Immunohistochemistry (IHC) showed positivity for epithelial membrane antigen (EMA) and somatostatin receptor 2 (SSTR2), and negativity for inhibin (**Figures 2B–D**). The Ki-67 proliferation index was low (5–10%) (**Figure 2E**). Based on these findings, the final pathological diagnosis was MM.

**Figure 3:**
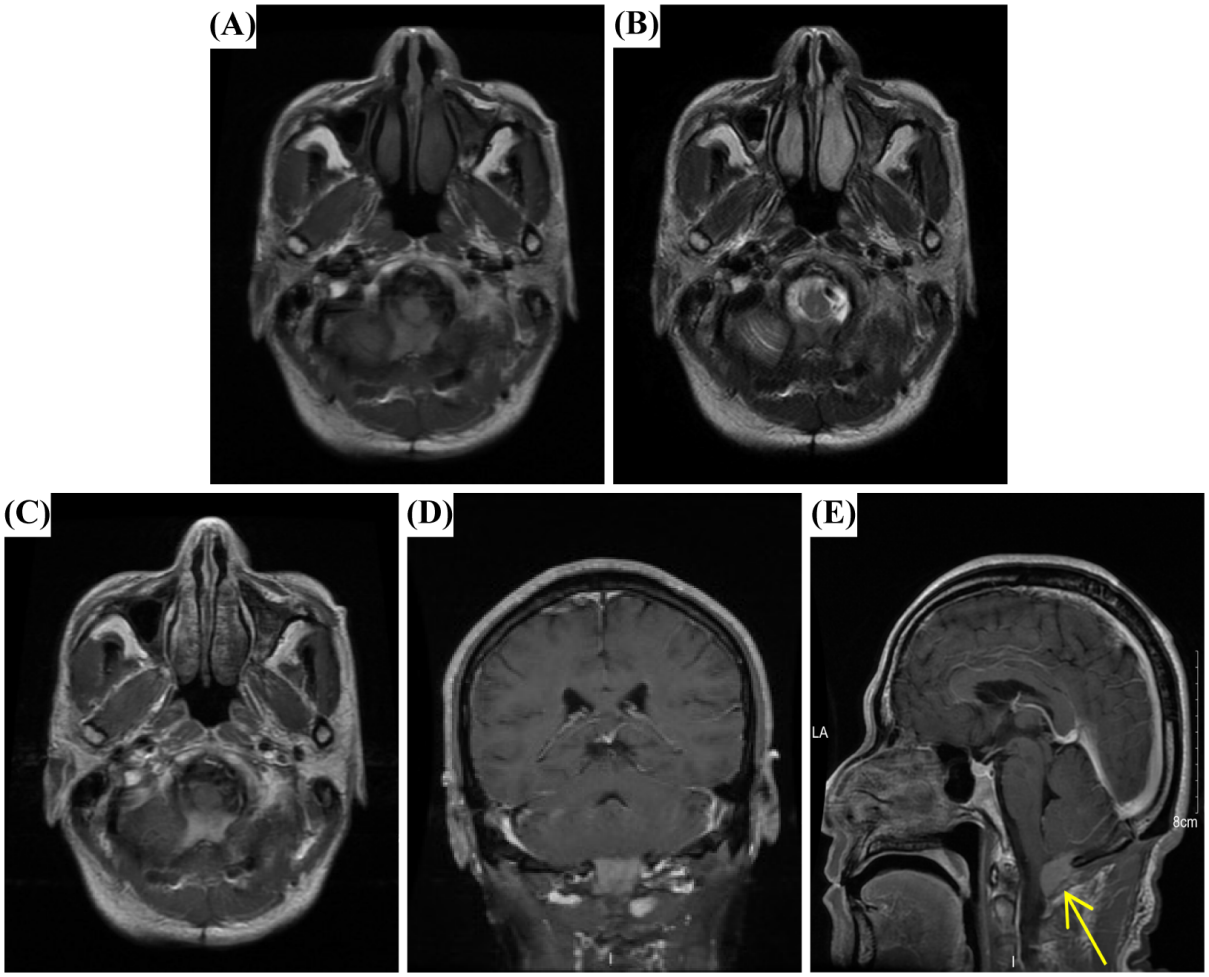
MRI findings of giant cell reparative granuloma (GCRG). **(A)** Axial T1WI revealed a flaky high-signal lesion in the posterior medulla oblongata at the surgical site. **(B)** Axial T2WI showed an iso–low signal lesion. **(C)** Diffusion-weighted imaging (DWI) demonstrated a slightly hyperintense cystic wall and hypointense content. **(D, E)** Post-contrast axial, coronal, and sagittal T1WI confirmed the presence of the mass (arrow).

Although the patient experienced mild dizziness and discomfort postoperatively, MRI performed three days after surgery confirmed complete tumor resection with no residual lesion. A six-month follow-up MRI at the local hospital showed no evidence of recurrence. However, a follow-up examination one year later revealed a mass with flaky high signal on T1WI (**Figure 3A**) and iso-low signal on T2WI (**Figure 3B**) in the posterior medulla oblongata at the surgical site. In addition, post-contrast axial, coronal, and sagittal T1WI further demonstrated the presence of the mass (**Figures 3C–E**), although the patient remained asymptomatic. This was considered a postoperative recurrence of the fourth ventricle tumor.

**Figure 4:**
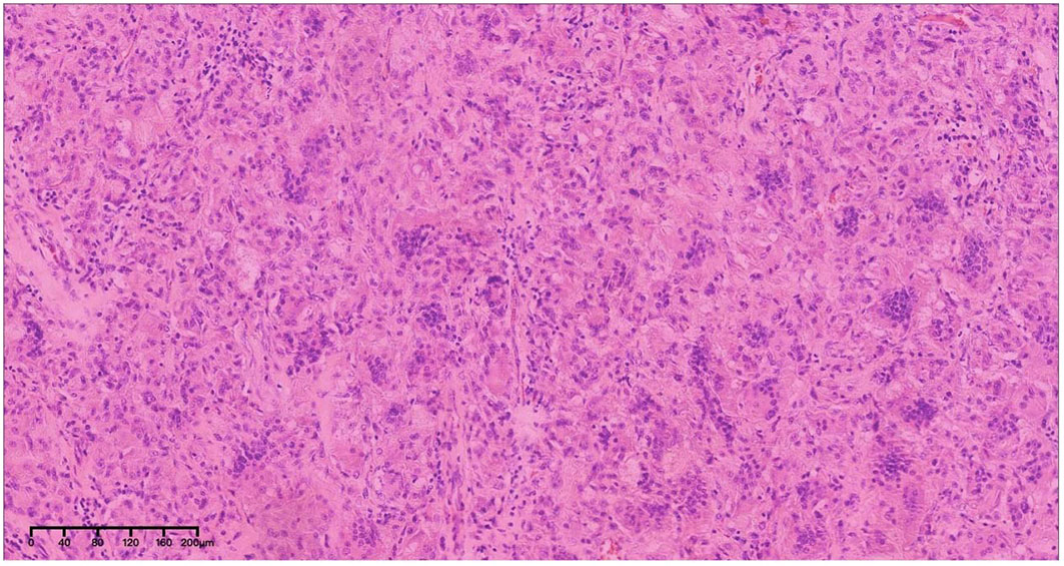
Pathological findings of GCRG. HE staining showed multiple aggregates of multinucleated giant cells, increased mononuclear-like cells, and interstitial fibrous tissue proliferation, forming a lesion rich in osteoblast-like giant cells.

After admission and exclusion of relevant contraindications, the space-occupying lesion was resected using the posterior median approach. During the operation, a tumor was identified extending from the inferior cerebellar margin to the inferior atlantoaxial margin. The tumor, measuring 2 × 3 cm, was firm, highly vascular, and densely adhered to the brainstem. Part of the tumor tissue was excised and sent for frozen section analysis, which revealed abundant of macrophages, consistent with granuloma formation. Further HE staining revealed multiple aggregates of multinucleated giant cells, an increased number of mononuclear-like cells, and interstitial fibrous tissue proliferation, consistent with a lesion rich in osteoblast-like giant cells (**Figure 4**). Considering the patient’s one-year prior surgical history and the histopathological findings on HE staining, the diagnosis favored GCRG. Postoperative cranial MRI confirmed complete tumor resection with no residual lesions. During subsequent follow-ups, the patient was examined locally, and no signs of recurrence were observed.

## Discussion

Meningiomas are the most common primary tumors of the central nervous system, arising from arachnoid cap or meningeal epithelial cells (4). They most commonly occur in the parafalcine, parasagittal, skull base, and convexity regions. In contrast, intraventricular meningiomas are rare, accounting for only 0.5%–3% of all meningiomas, with those in the fourth ventricle comprising just 5% of ventricular cases (5). Among meningioma subtypes, fibrous meningiomas are the most common, followed by transitional meningiomas, while MM represent only 1.6% of intracranial meningiomas (1). Here, we present an extremely rare case of MM located in the fourth ventricle, which, to our knowledge, has not been previously reported in humans. The diagnosis was confirmed through radiological and histopathological evaluation, and the lesion was successfully managed with complete surgical resection via a posterior median approach.

MM, a WHO grade I tumor, is a rare subtype of meningioma. On MRI, T1WI typically shows hypointense or mixed iso–hypointense signals, while T2WI reveals hyperintense or mixed iso–hyperintense signals. It is worth noting that after intravenous gadolinium-based contrast administration, the lesions often exhibit faint reticular or ring-like heterogeneous enhancement, and a dural tail sign is commonly observed (6). Additionally, Terada described a characteristic reticular enhancement pattern on T1WI and DWI, along with lower apparent diffusion coefficient (ADC) values in MM (7). The imaging findings in our case—particularly the mixed cystic components and dural tail sign—were partially consistent with these features and strongly suggested a diagnosis of MM.

Meningiomas in the fourth ventricle are exceptionally rare, making accurate differential diagnosis crucial. Due to the anatomical location, it is critical to distinguish meningiomas from other fourth ventricular tumors (8). Ependymomas, for instance, tend to have margins, may be solid or mildly cystic, are frequently accompanied by calcification, and are often associated with obstructive hydrocephalus—features less common in meningiomas (9). Medulloblastomas are highly malignant, often presenting irregular borders, heterogeneous enhancement, and significant cerebrospinal fluid flow obstruction, contrasting with the smoother, well-circumscribed appearance of meningiomas (10). Hemangioblastomas are characterized by intense contrast enhancement and prominent tumor-feeding vessels due to their rich vascularity, features rarely seen in meningiomas (11, 12). Compared to other solid tumors, such as gliomas or metastases, meningiomas are generally more circumscribed, less aggressive, and more likely to show calcification, which is uncommon in high-grade neoplasms (13, 14). Although initially diagnosed as a fourth ventricular ependymoma, the mixed cystic components and dural tail sign in our case distinguish it from ependymomas, which typically have a homogeneous structure and are associated with hydrocephalus. The heterogeneous enhancement observed, however, aligns more with MM.

MM exhibit distinct histopathologic features that help distinguish them from other types of meningiomas. Unlike typical meningiomas, which are generally more homogeneous, MM often show both microcystic and macrocystic changes, increased vascularity, and vitreous degeneration. These characteristics, along with a lower Ki-67 proliferation index, suggest that MM is less aggressive and exhibits lower mitotic activity compared to atypical or malignant meningiomas (15). MM also typically expresses EMA and SSTR2, which helps differentiate it from gliomas and germ cell tumors, both of which are usually EMA-negative (16, 17). These distinctive histopathological features facilitate the differentiation of MM from other meningioma subtypes and malignancies, contributing to a more favorable prognosis following complete resection.

In the present case, the patient underwent complete surgical resection of a fourth ventricular meningioma using the posterior median approach, a technique commonly employed for tumors in this region. This approach allows for direct access to the tumor while minimizing trauma to surrounding brain structures (18). The surgery was successful, and postoperative MRI confirmed the complete resection of the tumor, with no residual mass. Complete surgical resection of meningiomas, especially benign lesions, is associated with the lowest recurrence rates, while partial resection increases the likelihood of recurrence and often necessitates adjuvant treatments, such as radiotherapy, to manage residual disease (19, 20). In cases where complete resection is not feasible, or when the meningioma is atypical or malignant, adjuvant radiation therapy or stereotactic radiosurgery (SRS) is often employed. SRS is particularly indicated for smaller, localized tumors or when surgical resection carries a higher risk of damaging vital structures. Radiation therapy is also recommended for atypical meningiomas, which have a higher recurrence risk and may exhibit more aggressive behavior (21, 22). Postoperative surveillance is essential to monitor for recurrence, which can occur even after complete surgical resection, particularly in tumors with atypical histology or those located in challenging areas. In this case, we closely monitored the patient, and MRI performed six months postoperatively showed no signs of recurrence. Recurrence rates for completely resected meningiomas are generally low, with some studies reporting rates of less than 10% in benign cases (23). Long-term follow-up is recommended for all meningioma patients, typically involving regular imaging during the first few years after post-surgery.

However, at the one-year postoperative review, a GCRG was identified at the original surgical site. GCRG is a rare benign reactive lesion, typically arising in response to surgical trauma or tissue injury. It is characterized by macrophage aggregation, multinucleated giant cells, and chronic inflammation (24, 25). Although uncommon following brain surgery, its occurrence in this case is particularly noteworthy. Its development may have resulted from a delayed reactive inflammatory response secondary to surgical trauma. The hypervascularity of the initial resection and manipulation of brain tissue likely triggered this immune response, leading to granulomatous proliferation and ultimately the formation of GCRG. While GCRG may resemble tumor recurrence on imaging, it is pathologically distinct. This emphasizes the importance of distinguishing tumor recurrence from reactive lesions during postoperative surveillance, providing valuable insights into postoperative complications.

In summary, we present the first case of MM located in the fourth ventricle. MM is extremely rare in this region, and preoperative diagnosis can be challenging. MRI remains the most effective modality for early diagnosis. Surgical resection is the treatment of choice, with the final diagnosis confirmed by histopathological and IHC analysis. Notably, a GCRG that developed one year postoperatively was also successfully excised.

## Data Availability

The raw data supporting the conclusions of this article will be made available by the authors, without undue reservation.
